# Differential efficacy of Conbercept vs. Ranibizumab on retinal microstructural changes in macular edema secondary to retinal vein occlusion during the loading phase

**DOI:** 10.3389/fmed.2026.1885798

**Published:** 2026-08-12

**Authors:** Haiyue Yu, Zhijian Yao, Juan Teng, Qin Zhang, Tiangang Liu, Guang Zhu

**Affiliations:** 1 Department of Ophthalmology, The Second People's Hospital of Bengbu, Bengbu, Anhui, China; 2 Department of Ophthalmology, The Second Affiliated Hospital of Anhui Medical University, Hefei, Anhui, China

**Keywords:** Conbercept, disorganization of the retinal inner layers, optical coherence tomography, Ranibizumab, retinal vein occlusion

## Abstract

**Objective:**

To compare the efficacy of Conbercept vs.Ranibizumab on retinal microstructural changes in patients with retinal vein occlusion-associated macular edema (RVO-ME) by evaluating multidimensional OCT biomarkers during the loading phase.

**Methods:**

This retrospective cohort study analyzed treatment-naive RVO-ME patients receiving three monthly intravitreal injections of Conbercept or Ranibizumab. Propensity score matching (PSM) was used to balance baseline characteristics. Inter-group and intra-group comparisons of OCT biomarkers including central macular thickness (CMT), disorganization of the retinal inner layers ([DRIL), hyperreflective dots (HRD), intraretinal fluid (IRF), subretinal fluid (SRF), and ellipsoid zone (EZ) disruption] were performed using the Mann-Whitney *U*-test and the Friedman test. Generalized estimating equation (GEE) models were employed to evaluate longitudinal trends in CMT, DRIL, and IRF.

**Results:**

After PSM, 108 patients (54 per group) were analyzed. Both agents showed significant improvements in all OCT biomarkers and BCVA at months 1 and 3 compared to baseline (all *P* < 0.05). Inter-group comparisons revealed that Ranibizumab achieved significantly lower CMT at months 1 (*P* = 0.011) and 3 (*P* = 0.006), and lower IRF at month 1 (*P* = 0.008). Conversely, Conbercept demonstrated significantly lower DRIL at month 3 (*P* = 0.004). GEE analysis confirmed a significant treatment-time interaction for DRIL (*P* = 0.004), indicating a more favorable longitudinal recovery of inner retinal microstructure with Conbercept, whereas no significant differences were observed in the longitudinal trends of CMT or IRF improvement (*P* > 0.05).

**Conclusions:**

Conbercept and Ranibizumab showed comparable primary outcomes and subtle divergences in exploratory secondary OCT biomarkers during the loading phase: Conbercept yielded greater DRIL reduction, whereas Ranibizumab achieved lower CMT and IRF at early time points. These preliminary findings may generate hypotheses for an OCT phenotype-driven approach to precision anti-VEGF therapy in RVO-ME.

## 1 Introduction

Retinal vein occlusion (RVO) is the second most common retinal vascular disorder after diabetic retinopathy. Macular edema (ME) secondary to RVO is the primary cause of vision loss, significantly compromising patients' visual function and quality of life (1). Anti-vascular endothelial growth factor (anti-VEGF) therapy has been established as the standard of care, alleviating macular edema by inhibiting the VEGF signaling pathway. In clinical practice in China, both Conbercept and Ranibizumab are extensively employed and have demonstrated substantial efficacy. Nevertheless, treatment selection frequently depends on empirical judgment, underscoring a lack of evidence-based individualized strategies. Currently, efficacy assessment relies predominantly on central macular thickness (CMT) and best-corrected visual acuity (BCVA) (2). However, these conventional metrics do not fully capture retinal microstructural changes.

Recent studies suggest that specific optical coherence tomography (OCT) biomarkers—including outer retinal structural integrity, subretinal fluid status, and disorganization of the retinal inner layers (DRIL)—are closely correlated with visual prognosis (3, 4). Microstructural restoration provides an anatomical basis for functional recovery and may offer complementary information beyond conventional metrics such as CMT and BCVA. Conbercept (a recombinant fusion protein) and Ranibizumab (a monoclonal antibody fragment) differ in molecular structure and binding profiles, which may theoretically translate into distinct anatomical responses. While comparative evidence exists for conventional outcomes, direct comparative data focusing on multidimensional OCT-defined microstructural changes remain limited.

Therefore, this retrospective study utilized spectral-domain optical coherence tomography (SD-OCT) to evaluate patients with macular edema secondary to retinal vein occlusion (RVO-ME), aiming to quantitatively compare the differential effects of Conbercept vs. Ranibizumab on various OCT biomarkers during the loading phase. This specific observation window was selected to minimize confounding factors arising from heterogeneity in subsequent follow-up intervals, thereby enabling a more accurate assessment of the differential efficacy of the two agents on retinal microstructural changes during their peak pharmacokinetic phase. This study aims to explore the potential therapeutic profiles of each agent, providing preliminary insights to inform individualized treatment strategies for patients with RVO-ME.

## 2 Methods

### 2.1 Study population

This single-center, retrospective cohort study included patients with RVO-ME treated at our institution between November 2021 and May 2025. The inclusion criteria were as follows: (1) a primary diagnosis of RVO-ME [comprising both central retinal vein occlusion (CRVO) and branch retinal vein occlusion (BRVO), diagnosed based on standardized clinical and imaging criteria, including characteristic retinal hemorrhages and vascular tortuosity on fundus examination, and secondary macular edema confirmed via OCT] and being treatment-naïve to anti-VEGF therapy; and (2) receipt of intravitreal Conbercept or Ranibizumab injections and completion of a three-dose monthly loading phase. The exclusion criteria included: (1) a history of other concomitant vitreoretinal diseases or prior ocular surgery; (2) incomplete follow-up data at any key time point (defined as baseline, 1 month ± 7 days, or 3 months ± 14 days post-injection); (3) incomplete clinical records (specifically lacking BCVA or OCT images); and (4) poor OCT image quality (e.g., low signal strength or severe artifacts) precluding accurate retinal segmentation.

To ensure homogeneity of the evaluation, the analysis was strictly confined to the standard loading phase of anti-VEGF therapy. This selection strategy minimized confounding factors associated with heterogeneity in subsequent treatment regimens and irregular follow-up intervals, thereby enabling a precise assessment of the therapeutic efficacy of both agents on retinal microstructures during their peak pharmacological activity. This study adhered to the tenets of the Declaration of Helsinki. It was approved by the Ethics Committee of The Second People's Hospital of Bengbu (Approval No.: Ethical Review [2025] No. 2). Given the retrospective design and the de-identification of all patient data, the requirement for informed consent was waived.

### 2.2 Study protocol

All patients received three consecutive monthly intravitreal injections as a loading phase (with an injection interval of 30 ± 7 days): Conbercept (0.5 mg/0.05 mL) or Ranibizumab (0.5 mg/0.05 mL), according to the treating physician's routine clinical decision. During the 3-month loading phase, no medication switching or corticosteroid use occurred, and adjuvant retinal photocoagulation was performed only in the presence of extensive non-perfusion areas. Standardized ophthalmic examinations were performed at baseline, 1 month, and 3 months post-injection. The comprehensive examination protocol included best-corrected visual acuity (BCVA), intraocular pressure (IOP), slit-lamp biomicroscopy, indirect ophthalmoscopy, color fundus photography, and SD-OCT. BCVA was measured using the Standard Logarithmic Visual Acuity Chart and recorded in decimal notation; for statistical analysis, these values were converted to the logarithm of the minimum angle of resolution (LogMAR) using the equation: LogMAR = –log_10_ (decimal acuity).

OCT Image Acquisition and Quantitative Analysis: All OCT scans were obtained using the Heidelberg Spectralis SD-OCT system (Heidelberg Engineering, Heidelberg, Germany) with a standardized macular protocol (20° × 20° volume scan with 25 B-scans; ART = 9). The horizontal transfoveal B-scan through the foveal center was used for biomarker quantification, as cross-sectional retinal microstructure at the fovea correlates most strongly with visual function. Image analysis was performed independently by two experienced retinal specialists (associate chief physicians) masked to treatment assignment. The measured parameters included central macular thickness (CMT), the length of disorganization of the retinal inner layers (DRIL), the number of hyperreflective dots (HRD), the area of intraretinal fluid (IRF), the area of subretinal fluid (SRF), and the length of ellipsoid zone (EZ) disruption (5). Detailed definitions and measurement protocols for the various OCT biomarkers were based on our previously published protocol (Supplementary Table S1, Supplementary Figure S1, (31)). Assessment of Inter-grader Consistency: For all quantitative metrics, if the discrepancy between the two graders' measurements was < 10%, the mean value was used for analysis; if the discrepancy was ≥10%, the measurement was adjudicated by a third senior ophthalmologist, and the resulting consensus value was adopted.

The primary functional and anatomical outcomes were defined as the longitudinal trajectories of BCVA and CMT, respectively. Secondary outcomes included longitudinal changes in OCT microstructural biomarkers, including DRIL, HRD, IRF, SRF, and ellipsoid zone (EZ) disruption length.

### 2.3 Statistical analysis

All statistical analyses and data visualization were performed using the R statistical software (version 4.3.1). Continuous variables were assessed for normality using the Shapiro-Wilk test; normally distributed data were expressed as mean ± standard deviation (SD), while non-normally distributed data were presented as median (Q1, Q3). Categorical variables were reported as frequency and percentage [*n* (%)]. Propensity score matching (PSM) was used to balance baseline characteristics. To avoid excessive sample attrition, only variables with an initial standardized mean difference (SMD) > 0.2 were included in the matching model. Key clinical covariates (including age, sex, diagnosis type, disease duration, diabetes, and hypertension) were adjusted for in subsequent multivariate generalized estimating equation (GEE) models to control for residual confounding. Inter-group comparisons were conducted using the independent samples *t*-test, Mann-Whitney *U* test, or Chi-square (χ^2^) test, depending on the data distribution. The Friedman test was employed for intra-group comparisons across different time points, followed by *post-hoc* pairwise comparisons using the Wilcoxon signed-rank test with Bonferroni correction. These GEE models were employed to evaluate longitudinal trends for biomarkers exhibiting significant inter-group differences at specific time points. All tests were two-sided, with statistical significance defined as *P* < 0.05.

## 3 Results

### 3.1 Baseline characteristics

**Figure 1:**
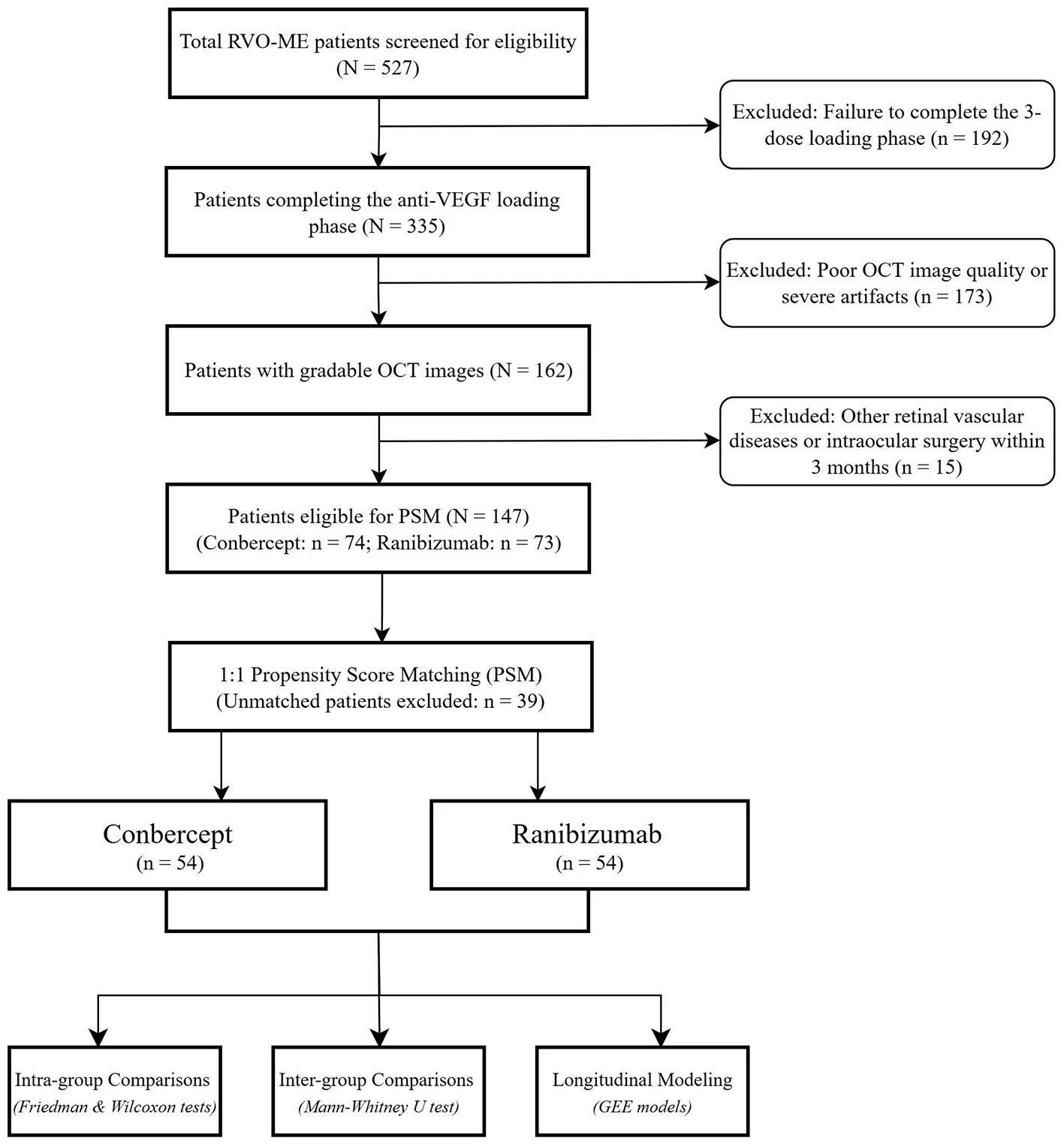
Flowchart of patient screening, enrollment, and propensity score matching.

A total of 527 patients with RVO-ME were initially screened for eligibility. Based on the predefined criteria, 380 patients were excluded (192 for failing to complete the 3-dose loading phase, 173 due to poor OCT image quality or severe artifacts, and 15 due to other retinal vascular diseases or recent intraocular surgery). Ultimately, 147 eligible patients were included for the initial analysis before propensity score matching (Conbercept group, *n* = 74; Ranibizumab group, *n* = 73) (Figure 1). Baseline characteristics of the excluded patients are detailed in Supplementary Table S2. While demographic parameters (age and sex) were comparable, the excluded cohort exhibited a higher proportion of CRVO (*P* = 0.008). To minimize selection bias, propensity score matching (PSM) was implemented. To avoid excessive sample attrition while maintaining bias control, the propensity score model targeted only baseline variables with SMD > 0.2 (CMT, IRF, diabetes, and hypertension). Matching was executed without replacement using the nearest-neighbor algorithm with a 1:1 ratio and a caliper width of 0.2.

**Figure 2:**
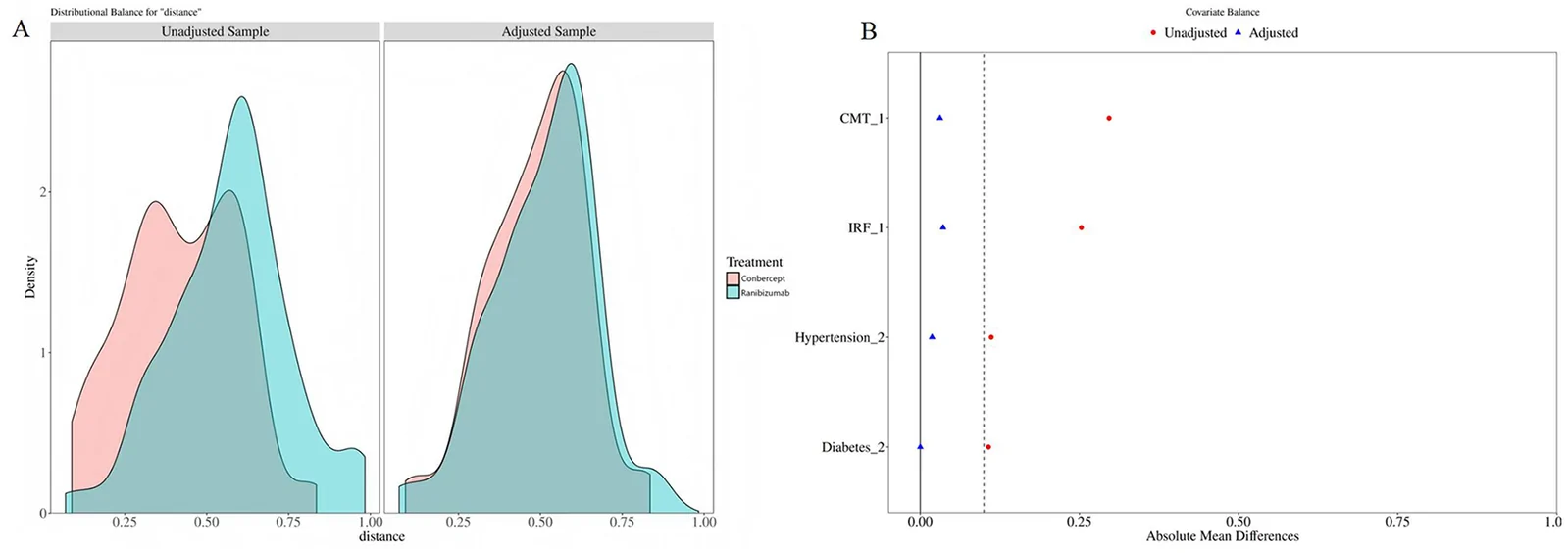
Balance of covariates before and after PSM. **(A)** Propensity score density plot. **(B)** Standardized mean difference (SMD) plot.

Ultimately, 108 patients were successfully matched and included in the analysis, with 54 patients in each group (Conbercept and Ranibizumab). Before matching, significant baseline imbalances were observed in central macular thickness (CMT) [*Z* = −2.462, *P* = 0.014] and diabetes history [χ^2^ = 3.929, *P* = 0.047]. Following PSM adjustment, all baseline characteristics were well-balanced between the groups (all SMD < 0.2, *P* > 0.05). The balance of covariate distribution before and after matching was visually verified using propensity score probability density plots and standardized mean difference (SMD) plots (Figure 2). Detailed baseline characteristics are presented in Table 1.

**Table 1:** Baseline characteristics of patients with RVO-ME before and after PSM.

Variable	Before PSM						After PSM					
	Total (*n* = 147)	Conbercept (*n* = 74)	Ranibizumab (*n* = 73)	Statistic	*P*	SMD	Total (*n* = 108)	Conbercept (*n* = 54)	Ranibizumab (*n* = 54)	Statistic	*P*	SMD
Age (years), Mean ± SD	59.90 ± 11.68	60.61 ± 11.45	59.19 ± 11.95	*t* = 0.734	0.464	−0.119	60.06 ± 11.35	61.39 ± 11.41	58.72 ± 11.24	*t* = 0.923	0.324	−0.157
Course (days), M (Q1, Q3)	30.00 (14.00, 60.00)	30.00 (10.00, 60.00)	30.00 (15.00, 60.00)	*Z* = −1.408	0.159	0.070	30.00 (14.00, 67.50)	30.00 (10.00, 82.50)	30.00 (15.00, 60.00)	*Z* = −0.452	0.651	0.060
BCVA, M (Q1, Q3)	0.92 (0.52, 1.30)	1.00 (0.63, 1.30)	0.82 (0.40, 1.30)	*Z* = −1.321	0.186	−0.123	0.92 (0.52, 1.24)	0.92 (0.60, 1.22)	0.76 (0.40, 1.28)	*Z* = −0.885	0.376	−0.075
CMT (μm), M (Q1, Q3)	547.00 (374.50, 850.00)	657.50 (434.75, 881.75)	473.00 (318.00, 785.00)	*Z* = −2.462	**0.014**	−0.297	507.00 (378.25, 775.50)	543.00 (387.25, 771.25)	482.50 (358.75, 776.75)	*Z* = −0.691	0.489	−0.033
DRIL (μm), M (Q1, Q3)	4,255.00 (2,676.50, 5,825.00)	4,758.50 (2,507.00, 5,858.00)	3,864.00 (2,798.00, 5,704.00)	*Z* = −1.100	0.272	−0.123	4,088.00 (2,275.50, 5,740.25)	4,192.00 (2,056.50, 5,838.25)	3,846.50 (2,441.75, 5,564.25)	*Z* = −0.169	0.866	−0.005
HRD, M (Q1, Q3)	44.00 (18.50, 76.50)	47.00 (23.00, 75.25)	38.00 (16.00, 77.00)	*Z* = −0.967	0.334	−0.083	36.50 (17.00, 70.50)	44.50 (23.25, 69.25)	29.50 (13.25, 74.25)	*Z* = −1.570	0.116	−0.126
SRF (mm^2^), M (Q1, Q3)	0.00 (0.00, 0.08)	0.00 (0.00, 0.14)	0.00 (0.00, 0.02)	*Z* = −1.724	0.085	−0.141	0.00 (0.00, 0.08)	0.00 (0.00, 0.09)	0.00 (0.00, 0.07)	*Z* = −0.459	0.647	0.075
IRF (mm^2^), M (Q1, Q3)	0.11 (0.03, 0.28)	0.14 (0.08, 0.29)	0.08 (0.00, 0.27)	*Z* = −1.675	0.094	0.253	0.11 (0.02, 0.27)	0.12 (0.06, 0.30)	0.08 (0.00, 0.22)	*Z* = −1.607	0.108	0.068
EZ Disruption Length (μm), M (Q1, Q3)	2,075.00 (595.00, 3,727.00)	2,143.00 (1,072.75, 3,776.50)	1,716.00 (133.00, 3,584.00)	*Z* = −1.027	0.304	−0.083	1,961.00 (580.75, 3,572.00)	1,961.00 (763.00, 3,573.75)	1,865.00 (200.50, 3,470.50)	*Z* = −0.296	0.768	0.041
Sex, *n* (%)				*χ^2^* = 0.007	0.936					*χ^2^* = 0.333	0.564	
Male	72 (48.98)	36 (48.65)	36 (49.32)			0.013	53 (49.07)	25 (46.30)	28 (51.85)			0.111
Female	75 (51.02)	38 (51.35)	37 (50.68)			−0.013	55 (50.93)	29 (53.70)	26 (48.15)			−0.111
Diagnosis, *n* (%)				*χ^2^* = 0.076	0.783					*χ^2^* = 0.045	0.832	
CRVO	56 (38.10)	29 (39.19)	27 (36.99)			−0.046	31 (28.7)	16 (29.63)	15 (27.78)			−0.041
BRVO	91 (61.90)	45 (60.81)	46 (63.01)			0.046	77 (71.3)	38 (70.37)	39 (72.22)			0.041
Diabetes, *n* (%)				*χ^2^* = 3.929	**0.047**					*χ^2^* = 0.000	1.000	
Yes	18 (12.24)	13 (17.57)	5 (6.85)			−0.424	10 (9.26)	5 (9.26)	5 (9.26)			0.000
No	129 (87.76)	61 (82.43)	68 (93.15)			0.424	98 (90.74)	49 (90.74)	49 (90.74)			0.000
Hypertension, *n* (%)				*χ^2^* = 2.960	0.085					*χ^2^* = 0.064	0.800	
Yes	119 (80.95)	64 (86.49)	55 (75.34)			−0.259	89 (82.41)	45 (83.33)	44 (81.48)			−0.048
No	28 (19.05)	10 (13.51)	18 (24.66)			0.259	19 (17.59)	9 (16.67)	10 (18.52)			0.048

Data are presented as mean ± standard deviation (SD), median (Q1, Q3), or number (percentage).BCVA, Best-corrected visual acuity; CMT, Central macular thickness; DRIL, Disorganization of the retinal inner layers; HRD, Hyperreflective dots; SRF, Subretinal fluid; IRF, Intraretinal fluid; EZ, Ellipsoid zone; PSM, Propensity score matching; SMD, Standardized mean difference. *t* was calculated by independent *t*-test; *Z* was calculated by the Mann-Whitney *U* test; χ^2^ was calculated by the Chi-square test. Bold values indicate *P* < 0.05.

### 3.2 Intra- and inter-group comparisons of BCVA and OCT biomarkers

**Figure 3:**
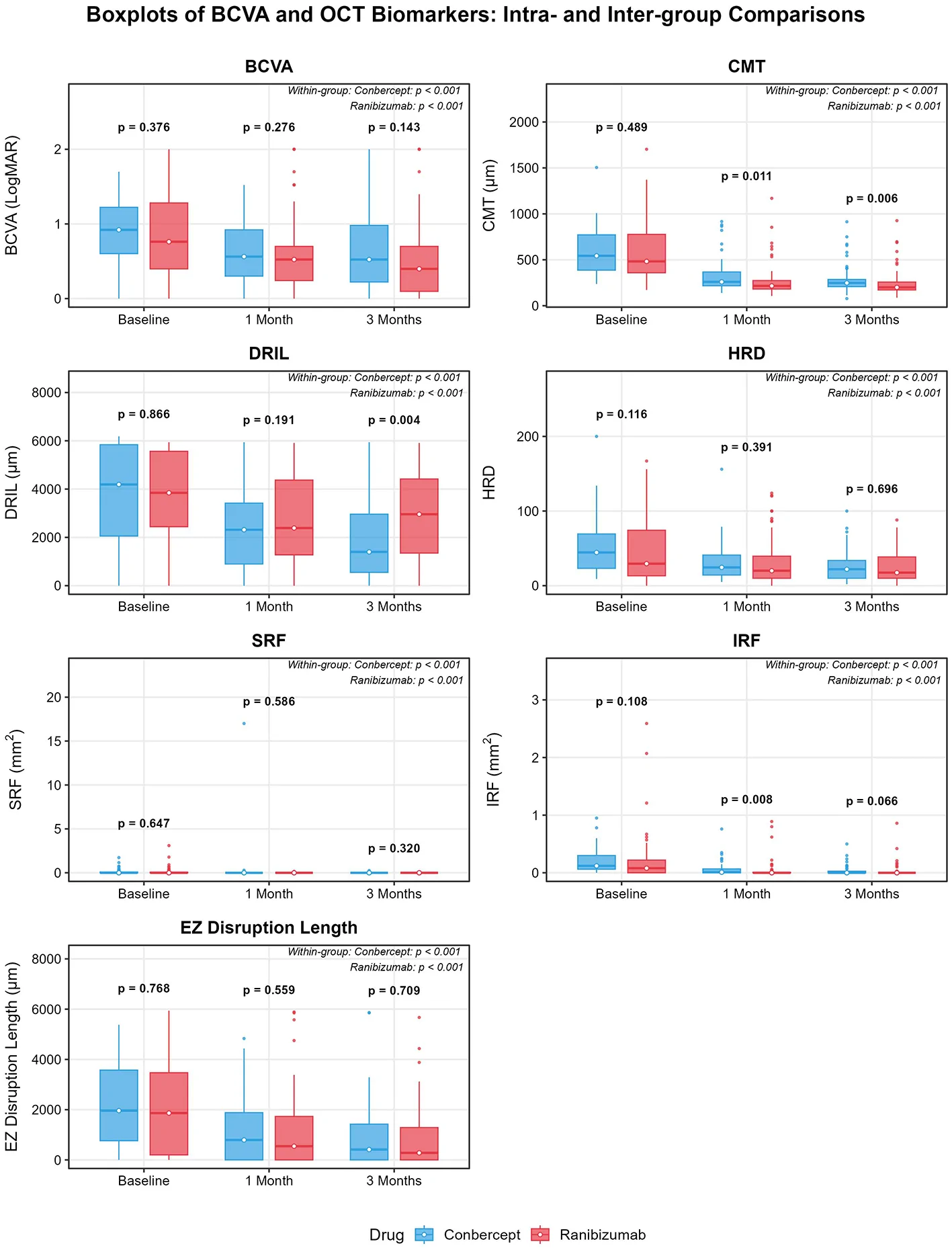
Boxplots of BCVA and OCT biomarkers: intra- and inter-group comparisons by drug group across follow-up time points.

Intra-group analyses using the Friedman test revealed significant improvements in all parameters compared with baseline in both the Conbercept and Ranibizumab groups (*P*<*0.05*). Notably, the median (IQR) of SRF area at baseline, 1 month, and 3 months was 0.00 (0.00, 0.09), 0.00 (0.00, 0.00), and 0.00 (0.00, 0.00) mm^2^ for Conbercept, and 0.00 (0.00, 0.07), 0.00 (0.00, 0.00), and 0.00 (0.00, 0.00) mm^2^ for Ranibizumab, respectively. The significant intra-group differences were driven by the complete resolution of fluid in patients with non-zero baseline SRF. Detailed pairwise comparisons across time points are provided in Supplementary Table S3. Regarding inter-group comparisons (Mann-Whitney *U* test), the Ranibizumab group exhibited significantly lower median CMT at 1 month (*P* = 0.011) and 3 months (*P* = 0.006), as well as lower IRF at 1 month (*P* = 0.008) compared to the Conbercept group. Conversely, the Conbercept group showed significantly reduced DRIL at 3 months compared to the Ranibizumab group (*P* = 0.004). No statistically significant differences were observed between the two groups regarding HRD, SRF, EZ disruption length, or BCVA at any follow-up time point (*P* > 0.05). These results are summarized in Table 2 and Figure 3.

**Table 2:** Intra-group and inter-group comparisons of BCVA and OCT biomarkers between Conbercept and Ranibizumab groups at baseline, 1 month, and 3 months.

Variable	Baseline	1 Month	3 Months	*χ^2^*	*P*
BCVA (LogMAR)					
Conbercept	0.92 (0.60, 1.22)	0.56 (0.30, 0.92)	0.52 (0.22, 0.98)	40.136	*p* < 0.001
Ranibizumab	0.76 (0.40, 1.28)	0.52 (0.24, 0.70)	0.40 (0.10, 0.70)	44.959	*p* < 0.001
Median Difference (95% CI)	0.10 (−0.10 to 0.30)	0.10 (−0.08 to 0.22)	0.12 (−0.00 to 0.30)		
*U*	1,602	1,635	1,695.5		
*P*	0.376	0.276	0.143		
CMT (μm)					
Conbercept	543.00 (387.25, 771.25)	259.50 (217.25, 366.75)	248.00 (208.25, 286.00)	65.833	*p* < 0.001
Ranibizumab	482.50 (358.75, 776.75)	216.00 (181.75, 273.75)	200.00 (172.00, 257.25)	61.598	*p* < 0.001
Median Difference (95% CI)	33.00 (−61.00 to 126.00)	40.00 (10.00 to 69.00)	41.00 (13.00 to 68.00)		
*U*	1,571	1,874	1,910		
*P*	0.489	0.011	0.006		
DRIL (μm)					
Conbercept	4,192.00 (2,056.50, 5,838.25)	2,315.50 (898.50, 3,418.75)	1,398.50 (552.00, 2,961.25)	40.170	*p* < 0.001
Ranibizumab	3,846.50 (2,441.75, 5,564.25)	2,388.00 (1,276.75, 4,373.25)	2,958.00 (1,350.00, 4,418.25)	22.365	*p* < 0.001
Median Difference (95% CI)	36.00 (−600.00 to 778.00)	−474.77 (−1,228.00 to 194.00)	−1,042.00 (−1,832.00 to −331.00)		
*U*	1,486	1,245	994		
*P*	0.866	0.191	0.004		
HRD					
Conbercept	44.50 (23.25, 69.25)	24.50 (14.25, 41.00)	22.00 (10.00, 33.75)	26.280	*p* < 0.001
Ranibizumab	29.50 (13.25, 74.25)	20.00 (10.00, 39.50)	17.50 (10.00, 38.50)	18.770	*p* < 0.001
Median Difference (95% CI)	8.00 (−2.00 to 20.00)	3.00 (−4.00 to 10.00)	1.00 (−5.00 to 8.00)		
*U*	1,714	1,598	1,522		
*P*	0.116	0.391	0.696		
SRF (mm^2^)					
Conbercept	0.00 (0.00, 0.09)	0.00 (0.00, 0.00)	0.00 (0.00, 0.00)	26.000	*p* < 0.001
Ranibizumab	0.00 (0.00, 0.07)	0.00 (0.00, 0.00)	0.00 (0.00, 0.00)	28.000	*p* < 0.001
Median Difference (95% CI)	0.00 (−0.00 to 0.00)	0.00 (−0.00 to 0.00)	0.00 (−0.00 to 0.00)		
*U*	1,524.5	1,516	1,511.5		
*P*	0.647	0.586	0.320		
IRF (mm^2^)					
Conbercept	0.12 (0.06, 0.30)	0.01 (0.00, 0.07)	0.00 (0.00, 0.03)	43.435	*p* < 0.001
Ranibizumab	0.08 (0.00, 0.22)	0.00 (0.00, 0.01)	0.00 (0.00, 0.00)	44.227	*p* < 0.001
Median Difference (95% CI)	0.03 (−0.00 to 0.08)	0.00 (0.00 to 0.02)	0.00 (−0.00 to 0.00)		
*U*	1,718.5	1,843	1,694.5		
*P*	0.108	0.008	0.066		
EZ Disruption Length (μm)					
Conbercept	1,961.00 (763.00, 3,573.75)	793.50 (0.00, 1,881.00)	413.00 (0.00, 1,422.75)	45.534	*p* < 0.001
Ranibizumab	1,865.00 (200.50, 3,470.50)	546.00 (0.00, 1,729.50)	280.50 (0.00, 1,287.25)	41.867	*p* < 0.001
Median Difference (95% CI)	48.38 (−649.00 to 721.00)	0.00 (−115.00 to 402.00)	0.00 (−0.00 to 94.00)		
*U*	1,506.5	1,552	1,516.5		
*P*	0.768	0.559	0.709		

BCVA, best-corrected visual acuity; CMT, central macular thickness; DRIL, disorganization of the retinal inner layers; HRD, hyperreflective dots; SRF, subretinal fluid; IRF, intraretinal fluid; EZ, ellipsoid zone. Data are expressed as median (Q1, Q3). Intra-group comparisons among different time points were performed using the Friedman test; Inter-group comparisons between the two treatment groups were performed using the Mann-Whitney *U* test. *P* < 0.05 was considered statistically significant.

To address the potential influence of numerical baseline variations, a supplementary analysis comparing absolute changes and percentage reductions from baseline was performed (Supplementary Table S4). Both groups demonstrated substantial absolute and relative reductions in CMT, DRIL, and IRF at month 3, but only for DRIL were significant differences observed (*P* < 0.001).

### 3.3 Evaluation of longitudinal trends in CMT, IRF, and DRIL via generalized estimating equations (GEE)

#### 3.3.1 Analysis of dynamic trends in CMT

After adjusting for covariates including age, sex, disease duration, and comorbidities, the interaction effect between treatment and follow-up time was not statistically significant (1 month: Wald χ^2^ = 1.010, P = 0.315; 3 months: Wald χ^2^ = 0.872, P = 0.350). This indicates that the longitudinal trend of CMT changes did not differ significantly between the Conbercept and Ranibizumab groups. Analysis of main effects revealed no significant difference in the drug group main effect (Wald χ^2^ = 0.120, *P* = 0.729), suggesting comparable overall CMT levels between the two agents. However, the main effect of time was statistically significant; compared to baseline, CMT significantly decreased at 1 month (β = −0.582, SE = 0.079, Wald χ^2^ = 54.314, *P* < 0.001) and showed a further reduction at 3 months (β = −0.694, SE = 0.085, Wald χ^2^ = 66.981, *P* < 0.001), demonstrating that both agents effectively reduced CMT over time.

**Figure 4:**
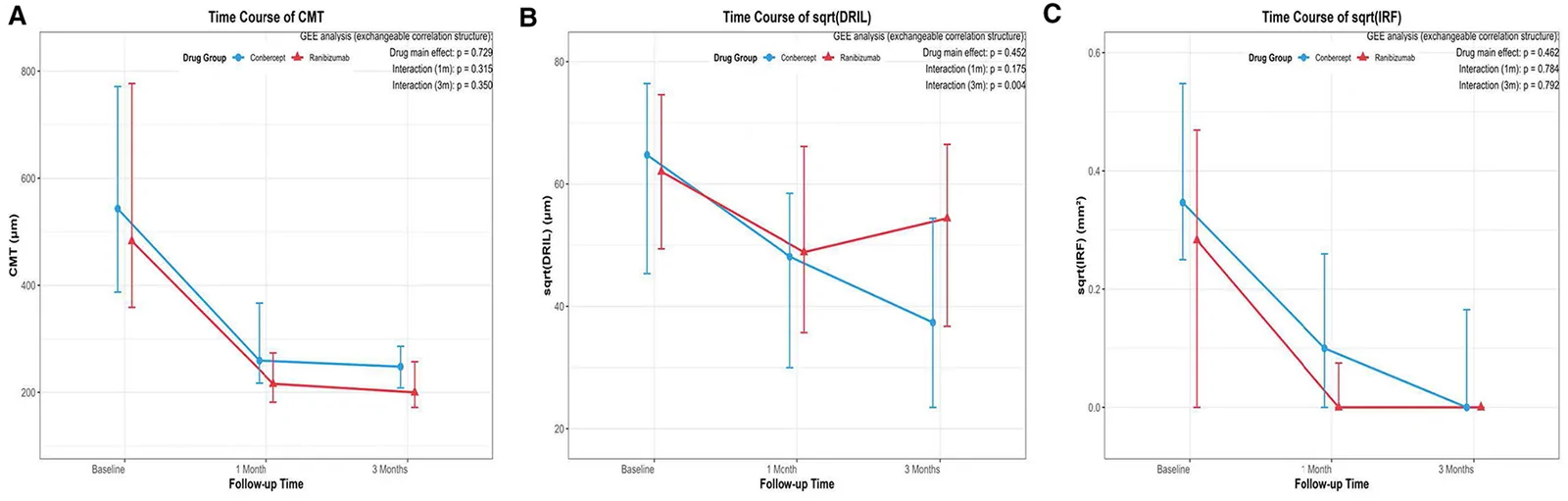
Time course of key OCT biomarkers by drug group across follow-up time points. **(A)** Time course of central macular thickness (CMT). **(B)** Time course of square-root transformed disorganization of retinal inner layers [sqrt(DRIL)]. **(C)** Time course of square-root transformed intraretinal fluid [sqrt(IRF)]. Blue lines represent the Conbercept group; red lines represent the Ranibizumab group. Error bars represent the interquartile range (IQR, 25th to 75th percentiles). The inset text shows *P*-values derived from GEE analysis for drug main effects and interactions at specific time points.

Furthermore, covariate analysis identified diagnosis type (BRVO vs. CRVO: Wald χ^2^ = 10.233, *P* = 0.001), disease duration (Wald χ^2^ = 8.487, *P* = 0.004), and diabetes status (Wald χ^2^ = 9.100, *P* = 0.003) as significant independent predictors of longitudinal CMT changes (Table 3 and Figure 4A).

**Table 3:** Factors influencing the dynamic changes in CMT analyzed by generalized estimating equations (GEE).

Variable	Estimate	Std. error	Wald *χ^2^*	*P*-value
(Intercept)	6.132	0.232	700.367	< 0.001
Drug (Ranibizumab vs. Conbercept)	−0.028	0.080	0.12	0.729
Time (1 Month vs. Baseline)	−0.582	0.079	54.314	< 0.001
Time (3 Months vs. Baseline)	−0.694	0.085	66.981	< 0.001
Age (years)	0.003	0.003	1.024	0.311
Sex (Female vs. Male)	−0.011	0.076	0.022	0.881
Diagnosis (BRVO vs. CRVO)	−0.279	0.087	10.233	0.001
Disease duration (days)	1.79 × 10^−4^	6.16 × 10^−5^	8.487	0.004
Diabetes (No vs. Yes)	0.267	0.089	9.100	0.003
Hypertension (No vs. Yes)	−0.087	0.088	0.993	0.319
Drug × Time (1 Month)	−0.120	0.120	1.010	0.315
Drug × Time (3 Months)	−0.117	0.125	0.872	0.350

CMT, central macular thickness; DRIL, disorganization of the retinal inner layers; HRD, hyperreflective dots; IRF, intraretinal fluid; SRF, subretinal fluid; EZ, ellipsoid zone. The GEE model adopted a Gamma distribution with a log link function, and an exchangeable correlation structure was used to handle the intra-subject correlation of repeated measurement data. Reference levels for categorical variables: Drug = Conbercept, Time = Baseline, Sex = Male, Diagnosis = CRVO, Diabetes = Yes, Hypertension = Yes.

#### 3.3.2 Analysis of dynamic trends in DRIL

Given that the raw DRIL data showed a slightly positively skewed distribution with mild residual trends, a square root transformation was applied for the GEE analysis. After adjusting for covariates including age, sex, disease duration, and comorbidities, analysis of the interaction between treatment and follow-up time revealed no significant interaction at 1 month (Wald χ^2^ = 1.843, *P* = 0.175). However, a significant interaction was observed at 3 months (Wald χ^2^ = 8.385, *P* = 0.004), indicating that the longitudinal trends of square-root transformed DRIL differed between the Conbercept and Ranibizumab groups, with divergence becoming apparent at 3 months. Although the overall main effect of the drug group was not statistically significant (Wald χ^2^ = 0.565, *P* = 0.452), the significant interaction term highlights that the therapeutic trajectories of the two agents were not parallel. The main effect of time was highly significant; compared to baseline, the square-root transformed DRIL decreased significantly at 1 month (β = −15.791, SE = 2.955, Wald χ^2^ = 28.557, *P* < 0.001) and showed a further reduction at 3 months (β = −21.611, SE = 2.970, Wald χ^2^ = 52.946, *P* < 0.001), demonstrating that both therapies effectively reduced DRIL severity over time.

For clinical clarity, GEE predictions were back-transformed to the original scale (μm). The adjusted marginal means of DRIL (95% CI) at Baseline, 1, and 3 months were 3,326.43 (2,598.04–4,144.70), 1,754.28 (1,191.12–2,426.13), and 1,300.62 (834.98–1,869.02) for Conbercept, and 3,596.83 (2,987.41–4,262.78), 2,441.00 (1,870.55–3,087.25), and 2,481.88 (1,881.50–3,165.27) for Ranibizumab, respectively.

Furthermore, covariate analysis identified age (Wald χ^2^ = 28.407, *P* < 0.001), diagnosis type (BRVO vs. CRVO: Wald χ^2^ = 32.012, *P* < 0.001), disease duration (Wald χ^2^ = 19.093, *P* < 0.001), and diabetes status (Wald χ^2^ = 4.250, *P* = 0.039) as significant independent predictors of longitudinal changes in square-root transformed DRIL (Table 4 and Figure 4B).

**Table 4:** Factors influencing the dynamic changes in square-root transformed DRIL analyzed by generalized estimating equations (GEE).

Variable	Estimate	Std. error	Wald *χ^2^*	*P*-value
(Intercept)	19.980	8.996	4.933	0.026
Drug (Ranibizumab vs. Conbercept)	2.298	3.057	0.565	0.452
Time (1 Month vs. Baseline)	−15.791	2.955	28.557	< 0.001
Time (3 Months vs. Baseline)	−21.611	2.970	52.946	< 0.001
Age (years)	0.705	0.132	28.407	< 0.001
Sex (Female vs. Male)	−1.710	3.083	0.308	0.579
Diagnosis (BRVO vs. CRVO)	−16.119	2.849	32.012	< 0.001
Disease duration (days)	0.007	0.002	19.093	< 0.001
Diabetes (No vs. Yes)	8.083	3.921	4.250	0.039
Hypertension (No vs. Yes)	−1.461	3.822	0.146	0.702
Drug × Time (1 Month)	5.224	3.848	1.843	0.175
Drug × Time (3 Months)	11.456	3.956	8.385	0.004

CMT, central macular thickness; DRIL, disorganization of the retinal inner layers; HRD, hyperreflective dots; IRF, intraretinal fluid; SRF, subretinal fluid; EZ, ellipsoid zone. The dependent variable of the GEE model was the square-root of repeated-measured DRIL. DRIL showed a slightly positively skewed distribution with mild residual trends; after square-root transformation, residuals exhibited no obvious trend. The GEE model adopted a Gaussian distribution with an identity link function, and an exchangeable correlation structure was used to handle the intra-subject correlation of repeated measurement data. Reference levels for categorical variables: Drug = Conbercept, Time = Baseline, Sex = Male, Diagnosis = CRVO, Diabetes = Yes, Hypertension = Yes.

#### 3.3.3 Analysis of dynamic trends in IRF

The raw IRF showed a positively skewed distribution with mild residual trends; however, after square-root transformation, residuals exhibited no obvious trend. After adjusting for covariates including age, sex, disease duration, and comorbidities, the interaction effect between treatment and follow-up time was not statistically significant (1 month: Wald χ^2^ = 0.075, *P* = 0.784; 3 months: Wald χ^2^ = 0.070, *P* = 0.792). This indicates that the longitudinal trends of square-root transformed IRF did not differ significantly between the Conbercept and Ranibizumab groups. Analysis of main effects revealed no significant difference in the drug group main effect (Wald χ^2^ = 0.541, *P* = 0.462), suggesting comparable overall levels of square-root transformed IRF between the two agents. However, the main effect of time was highly significant; compared to baseline, the square-root transformed IRF decreased significantly at 1 month (β = −0.215, SE = 0.032, Wald χ^2^ = 44.694, *P* < 0.001) and showed a further reduction at 3 months (β = −0.273, SE = 0.032, Wald χ^2^ = 72.379, *P* < 0.001), demonstrating that both therapies effectively reduced square-root transformed IRF over time.

For clinical clarity, GEE predictions were back-transformed to the original scale (mm^2^). The adjusted marginal means of IRF (95% CI) at Baseline, 1, and 3 months were 0.14 (0.09–0.20), 0.03 (0.01–0.05), and 0.01 (0.00–0.03) for Conbercept, and 0.10 (0.05–0.16), 0.01 (0.00–0.02), and 0.00 (0.00–0.01) for Ranibizumab, respectively.

Furthermore, covariate analysis identified diagnosis type (Wald χ^2^ = 6.950, *P* = 0.008) as a significant independent predictor of longitudinal changes in square-root transformed IRF. Meanwhile, diabetes status (Wald χ^2^ = 3.831, *P* = 0.050) and disease duration (Wald χ^2^ = 3.771, *P* = 0.052) exhibited borderline marginal trends influencing the IRF resolution trajectory (Table 5 and Figure 4C).

**Table 5:** Factors influencing the dynamic changes in square-root transformed IRF analyzed by generalized estimating equations (GEE).

Variable	Estimate	Std. error	Wald *χ^2^*	*P*-value
(Intercept)	0.303	0.106	8.163	0.004
Drug (Ranibizumab vs. Conbercept)	−0.041	0.056	0.541	0.462
Time (1 Month vs. Baseline)	−0.215	0.032	44.694	< 0.001
Time (3 Months vs. Baseline)	−0.273	0.032	72.379	< 0.001
Age (years)	0.001	0.001	1.192	0.275
Sex (Female vs. Male)	−0.019	0.032	0.333	0.564
Diagnosis (BRVO vs. CRVO)	−0.110	0.042	6.950	0.008
Disease duration (days)	8.21 × 10^−5^	4.23 × 10^−5^	3.771	0.052
Diabetes (No vs. Yes)	0.072	0.037	3.831	0.050
Hypertension (No vs. Yes)	−0.026	0.038	0.473	0.492
Drug × Time (1 Month)	−0.016	0.060	0.075	0.784
Drug × Time (3 Months)	0.014	0.054	0.070	0.792

CMT, central macular thickness; DRIL, disorganization of the retinal inner layers; HRD, hyperreflective dots; IRF, intraretinal fluid; SRF, subretinal fluid; EZ, ellipsoid zone. The dependent variable of the GEE model was the square-root of repeated-measured IRF. IRF showed a positively skewed distribution with mild residual trends; after square-root transformation, residuals exhibited no obvious trend. The GEE model adopted a Gaussian distribution with an identity link function, and an exchangeable correlation structure was used to handle the intra-subject correlation of repeated measurement data. Reference levels for categorical variables: Drug = Conbercept, Time = Baseline, Sex = Male, Diagnosis = CRVO, Diabetes = Yes, Hypertension = Yes.

### 3.4 Safety and adverse events

No ocular or systemic adverse events (including infectious endophthalmitis, sterile intraocular inflammation, persistent IOP elevation, or other systemic complications) were observed in either group during the 3-month loading phase.

## 4 Discussion

Macular edema (ME) is the leading cause of vision loss in retinal vein occlusion (RVO), for which anti-VEGF therapy is the standard of care. However, the lack of specific OCT phenotype-based guidance limits individualized treatment. Utilizing PSM and longitudinal analysis, this retrospective study compared the microstructural restorative efficacy of Conbercept vs. Ranibizumab over a 3-month loading phase. While both agents significantly improved visual acuity and retinal anatomy, they exhibited subtle morphological divergences: Conbercept yielded greater DRIL reduction, whereas Ranibizumab achieved lower early CMT and IRF, despite comparable overall edema resolution. These findings reaffirm anti-VEGF efficacy and suggest that integrating OCT phenotypes may offer preliminary insights for tailored therapy.

Intragroup comparisons using non-parametric repeated measures analysis revealed significant improvements in CMT, DRIL, HRD, SRF, IRF, EZ disruption length, and BCVA at 1 and 3 months post-treatment compared to baseline in both cohorts (all intragroup *P*<*0.05*). Consistent with previous reports (6), these findings further confirm that both Conbercept and Ranibizumab effectively ameliorate a spectrum of OCT biomarkers closely associated with retinal structural integrity and visual function. Specifically, the resolution of edema-related parameters (CMT, IRF, and SRF) signifies the structural restoration of inner and outer retinal barriers, constituting the anatomical foundation for visual improvement (3, 4, 7, 8); meanwhile, improvements in DRIL and HRD morphologically reflect improved inner retinal microcirculation and alleviated local inflammatory responses (9–12). Furthermore, the dynamic reduction in EZ disruption reflects the restoration of photoreceptor structural continuity, providing the essential structural prerequisite for visual signal transduction (13–15). Taken together, by dynamically tracking multidimensional OCT biomarkers, this study systematically validates that both agents promote retinal anatomical restoration, indirectly implying their clinical value in preserving the structural integrity of the retinal neurovascular unit.

Although cross-sectional intergroup comparisons revealed that the median CMT in the Ranibizumab group was significantly lower than in the Conbercept group at 1 and 3 months post-treatment (*P* = 0.011 and *P* = 0.006, respectively), and IRF was significantly lower at 1 month post-treatment (*P* = 0.008), GEE analysis indicated no statistically significant differences in the longitudinal trends of improvement for either parameter (interaction effects *P* > 0.05). This suggests that while overall fluid resolution profiles are parallel, Ranibizumab exhibited lower absolute edema parameters during the initial phase. VEGF-A is a critical mediator of neovascularization and vascular leakage (16). Both Conbercept and Ranibizumab inhibit the binding of VEGF-A to VEGFR1 and VEGFR2, thereby reducing angiogenesis and leakage to achieve comparable therapeutic outcomes (17–19). However, this initial response is primarily governed by molecular weight; Ranibizumab's smaller molecular size may facilitate faster vitreous diffusion and tissue penetration, enabling the rapid neutralization of VEGF-A (20, 21). Nevertheless, as intraocular pharmacokinetic parameters were not evaluated in this study, this physical mechanism remains a speculative explanation.

At 3 months post-treatment, DRIL in the Conbercept group was significantly lower than in the Ranibizumab group (*P* = 0.004). Further GEE analysis confirmed a significant treatment-time interaction (*P* = 0.004), indicating that Conbercept demonstrated a more pronounced longitudinal effect in the resolution of DRIL. This distinct advantage may be attributed to the multi-target mechanism of Conbercept, which simultaneously targets VEGF-A, VEGF-B, and placental growth factor (PlGF) (22). Although VEGF-B plays a limited role in the initiation of neovascularization, it regulates the expression of multiple vascular pro-survival genes via Neuropilin-1 (NP-1) and VEGFR-1, thereby protecting nascent vessels from apoptosis (23). Meanwhile, PlGF is primarily implicated in pathological retinal angiogenesis and inflammation (24). Furthermore, PlGF acts synergistically with VEGF to promote blood-retinal barrier (BRB) breakdown by activating the NF-κB pathway (25). The resolution of DRIL is generally regarded as an indicator of the stabilization of the inner retinal microenvironment and the restoration of glial cell function. Thus, we hypothesize that Conbercept, through its multi-target inhibition, may promote vascular repair and alleviate inflammatory responses more comprehensively, potentially providing a superior microenvironment for the retinal neurovascular unit, which may morphologically manifest as the significant resolution of DRIL. Consistent with this hypothesis, recent studies have demonstrated that anti-VEGF therapy with Ranibizumab and Conbercept induces dynamic retinal vascular morphological remodeling (26, 27). Although these studies primarily described vascular changes rather than DRIL, these findings may suggest that anti-VEGF therapy might also promote distinct retinal structural reorganization. However, these explanations remain speculative and require confirmation through future aqueous humor analysis and prospective microvascular imaging.

Compared with previous studies, our findings showed comparable efficacy between the two agents, whereas some literature reported that Conbercept was superior in BCVA and CMT improvement for RVO-ME (28, 29). This discrepancy may be attributed to the relatively short observation period of our study, during which significant differences in these outcomes may not have fully manifested. It is noteworthy that, although this study demonstrated no significant difference in BCVA between the two agents during the loading phase, the clinical relevance of differential DRIL resolution warrants consideration. While BCVA is primarily dependent on the structural integrity of the EZ, the retinal ischemia and structural disorganization reflected by DRIL serve not only as critical predictors of final visual acuity but are also closely correlated with visual quality (9, 12, 30).

Consequently, dynamically evaluating OCT biomarkers revealed potential differences in microstructural responses: Ranibizumab exhibited lower early edema, whereas Conbercept showed greater DRIL reduction. These findings suggest the value of incorporating OCT biomarkers into routine RVO management, cautiously supporting the exploration of OCT-guided individualized intervention. Specifically, Ranibizumab may offer advantages for rapid edema resolution to achieve vision salvage in cases presenting with high CMT or prominent IRF; conversely, Conbercept may be more favorable for promoting inner retinal microstructural restoration in cases characterized by extensive DRIL.

This study's robustness relies on PSM to balance baseline characteristics and minimize retrospective selection bias, alongside GEE models to precisely capture longitudinal microstructural dynamics. However, several limitations warrant acknowledgment. First, the retrospective design introduces selection and attrition biases; our strict eligibility criteria resulted in a high exclusion rate, with a higher proportion of patients with CRVO in the excluded cohort, which limits the external validity of our findings. Additionally, matched sample size constraints precluded separate CRVO and BRVO subgroup analyses. Second, despite double-masked grading and adjudication to minimize systematic errors, manual quantification of OCT biomarkers inevitably introduces subjectivity. Furthermore, a single foveal-centered B-scan cannot fully capture the 3D volumetric distribution of IRF and SRF, which may lead to incomplete assessment of overall macular edema burden. Third, baseline fluorescein angiography (FA) and OCT angiography (OCTA) were not performed routinely, limiting the objective assessment of macular perfusion and ischemia, which are closely associated with DRIL. Fourth, omitting certain baseline variables from the PSM model to preserve sample size may leave clinically relevant residual confounding. Furthermore, unmeasured socioeconomic factors, such as patient out-of-pocket costs and local insurance policies, could influence real-world drug selection. Finally, the 3-month follow-up limits the evaluation of long-term outcomes. However, this design deliberately circumvents confounding from heterogeneous subsequent regimens (e.g., PRN or T&E), ensuring a precise evaluation of microstructural changes during peak pharmacological activity. Future investigations should integrate AI-assisted analysis into multicenter, prospective, long-term studies to validate these differential efficacy profiles and elucidate the underlying mechanisms, thereby establishing a robust evidence base for the precision treatment of RVO.

In conclusion, Conbercept and Ranibizumab showed comparable primary outcomes (BCVA and CMT) and subtle divergences in exploratory secondary OCT biomarkers during the loading phase: Conbercept yielded greater DRIL reduction, whereas Ranibizumab achieved lower CMT and IRF at early cross-sectional time points. Collectively, these exploratory findings suggest that integrating multidimensional OCT features may be useful for hypothesis generation regarding individualized treatment decisions. However, given the retrospective design, these preliminary results cannot establish a definitive clinical framework and warrant further validation through prospective randomized trials.

## Data Availability

The raw data supporting the conclusions of this article will be made available by the authors, without undue reservation.
