# Supplementary material for: Differential efficacy of Conbercept vs. Ranibizumab on retinal microstructural changes in macular edema secondary to retinal vein occlusion during the loading phase
Source: Front Med (Lausanne). 2026 Aug 12;13:1885798. doi: 10.3389/fmed.2026.1885798 (PMC13506388; doi:10.3389/fmed.2026.1885798)
Supplement: Supplementary file 1 [file Table_1.docx]

**Supplementary Material**

Table S1 **Definitions and standardized measurement methods of each OCT biomarker**

| Biomarker | **Definitions** | **Standardized measurement methods** |
| --- | --- | --- |
| Central macular thickness (CMT) | The distance from the internal limiting membrane to the inner surface of the retinal pigment epithelium at the foveal level | CMT was measured using the built-in software of the OCT device. |
| The length of disorganization of the retinal inner layers (DRIL) | The state in which the boundary between any two of the ganglion cell-inner plexiform layer complex, inner nuclear layer, and outer plexiform layer cannot be identified within the horizontal OCT scan range | The length of DRIL was assessed across the full 6-mm width of the horizontal transfoveal B-scan at the foveal level using the built-in manual caliper tool. When DRIL was discontinuous, the lengths of all affected segments were summed. |
| The number of hyperreflective dots (HRD) | Small hyperreflective foci characterized by clear boundaries and a diameter of 20–40 μm | Manually counted using ImageJ across the full retinal thickness from the internal limiting membrane (ILM) to the retinal pigment epithelium (RPE), spanning the entire 6-mm horizontal width centered on the fovea. |
| The cross-sectional area of intraretinal fluid (IRF) | Accumulation of fluid within retinal layers (typically accompanied by retinal thickening), identified as hyporeflective spaces on OCT scans | The maximum cross-sectional area of IRF at the foveal level was measured using the built-in software of the OCT device. |
| The cross-sectional area of subretinal fluid (SRF) | Accumulation of fluid located between the outer surface of the photoreceptors and the inner surface of the retinal pigment epithelium (RPE) | The maximum cross-sectional area of SRF at the foveal level was measured using the built-in software of the OCT device. |
| The length of ellipsoid zone (EZ) disruption | Discontinuity or loss of the continuous hyperreflective EZ band on OCT foveal scans. | The length of EZ disruption at the foveal level was measured directly using the built-in software of the OCT device; if the EZ was completely continuous, the length was recorded as 0 μm. |

Table S2. Comparison of baseline characteristics between the included cohort and the excluded cohort.

| Characteristic | level | Included Cohort | Excluded cohort | P value |
| --- | --- | --- | --- | --- |
| n |  | 147 | 380 |  |
| Age, years (mean (SD)) |  | 59.90 (11.68) | 60.62 (11.23) | 0.514 |
| Sex (%) | Male | 72 (48.98) | 189 (49.74) | 0.881 |
|  | Female | 75 (51.02) | 191 (50.26) |  |
| Diagnosis (%) | CRVO | 56 (38.10) | 194 (51.05) | 0.008 |
|  | BRVO | 91 (61.90) | 186 (48.95) |  |

Table S3 Post-hoc pairwise intra-group comparisons of outcomes across time points (Friedman test followed by Wilcoxon signed-rank test with multiple comparisons adjustment)

| Group | Outcome | Time point comparison | statistic | P.adj |
| --- | --- | --- | --- | --- |
| Conbercept | BCVA | Baseline vs 1 Month | 980.5 | 0.000000531 |
|  | BCVA | Baseline vs 3 Months | 1040 | 0.0000648 |
|  | BCVA | 1 Month vs 3 Months | 270 | 1 |
|  | CMT | Baseline vs 1 Month | 1408 | 0.0000000309 |
|  | CMT | Baseline vs 3 Months | 1435 | 0.00000000765 |
|  | CMT | 1 Month vs 3 Months | 1029.5 | 0.017 |
|  | DRIL | Baseline vs 1 Month | 1127 | 0.00000705 |
|  | DRIL | Baseline vs 3 Months | 1200 | 0.00000148 |
|  | DRIL | 1 Month vs 3 Months | 853.5 | 0.02 |
|  | HRD | Baseline vs 1 Month | 1226.5 | 0.0000939 |
|  | HRD | Baseline vs 3 Months | 1229.5 | 0.000084 |
|  | HRD | 1 Month vs 3 Months | 963 | 0.038 |
|  | SRF | Baseline vs 1 Month | 197 | 0.015 |
|  | SRF | Baseline vs 3 Months | 259 | 0.000738 |
|  | SRF | 1 Month vs 3 Months | 45 | 0.027 |
|  | IRF | Baseline vs 1 Month | 1133.5 | 0.000000669 |
|  | IRF | Baseline vs 3 Months | 1172.5 | 0.000000078 |
|  | IRF | 1 Month vs 3 Months | 338.5 | 0.027 |
|  | EZ_Disruption_Length | Baseline vs 1 Month | 1096 | 0.00000465 |
|  | EZ_Disruption_Length | Baseline vs 3 Months | 1135 | 0.00000483 |
|  | EZ_Disruption_Length | 1 Month vs 3 Months | 552 | 0.172 |
| Ranibizumab | BCVA | Baseline vs 1 Month | 829.5 | 0.00000702 |
|  | BCVA | Baseline vs 3 Months | 913.5 | 0.00000321 |
|  | BCVA | 1 Month vs 3 Months | 424 | 0.465 |
|  | CMT | Baseline vs 1 Month | 1427 | 0.0000000116 |
|  | CMT | Baseline vs 3 Months | 1404 | 0.00000000336 |
|  | CMT | 1 Month vs 3 Months | 1020 | 0.021 |
|  | DRIL | Baseline vs 1 Month | 1050 | 0.0000414 |
|  | DRIL | Baseline vs 3 Months | 1093 | 0.00017 |
|  | DRIL | 1 Month vs 3 Months | 502 | 1 |
|  | HRD | Baseline vs 1 Month | 1027 | 0.044 |
|  | HRD | Baseline vs 3 Months | 1148.5 | 0.001 |
|  | HRD | 1 Month vs 3 Months | 885 | 0.113 |
|  | SRF | Baseline vs 1 Month | 220 | 0.000897 |
|  | SRF | Baseline vs 3 Months | 223 | 0.000597 |
|  | SRF | 1 Month vs 3 Months | 40 | 0.127 |
|  | IRF | Baseline vs 1 Month | 697 | 0.0000567 |
|  | IRF | Baseline vs 3 Months | 733 | 0.00000525 |
|  | IRF | 1 Month vs 3 Months | 73 | 0.627 |
|  | EZ Disruption Length | Baseline vs 1 Month | 839 | 0.00000393 |
|  | EZ Disruption Length | Baseline vs 3 Months | 913 | 0.000000333 |
|  | EZ Disruption Length | 1 Month vs 3 Months | 528.5 | 0.023 |

Table S4. Changes in CMT, DRIL, and IRF at month 3.

| Parameter | Conbercept (n = 54) | Ranibizumab (n = 54) | *P* value |
| --- | --- | --- | --- |
| ΔCMT (μm) | 238.50 (122.25, 407.50) | 262.50 (119.50, 442.00) | 0.946 |
| ΔCMT (%) | 46.56 (28.77, 67.98) | 54.74 (23.48, 67.47) | 0.474 |
| ΔDRIL (μm) | 1730.00 (654.00, 3531.00) | 718.00 (0.00, 1539.25) | < 0.001 |
| ΔDRIL (%) | 61.74 (29.73, 80.92) | 21.83 (0.00, 43.28) | < 0.001 |
| ΔIRF (mm²) | 0.11 (0.04, 0.27) | 0.08 (0.00, 0.22) | 0.283 |

Note: Data are expressed as median (interquartile range). Δ represents the reduction from baseline to month 3. Group differences were analyzed using the Wilcoxon rank-sum test. CMT, central macular thickness; DRIL, disorganization of the retinal inner layers; IRF, intraretinal fluid.

Supplementary Figure S1 Representative OCT images of biomarkers and standardized measurement protocols.

**(a)** Central Macular Thickness (CMT): Measurement of the distance between the internal limiting membrane (ILM) and the inner surface of the retinal pigment epithelium (RPE) at the foveal center.

**(b)** Disorganization of the Retinal Inner Layers (DRIL): Illustration of the horizontal extent where the boundaries between the inner retinal layers are indistinguishable.

**(c)** Hyperreflective Dots (HRD): Presence of small, discrete hyperreflective foci (yellow arrows) with diameters ranging from 20 to 40 μm.

**(d)** Intraretinal Fluid (IRF): Quantitative assessment of the maximum cross-sectional area of intraretinal fluid at the foveal level.

**(e)** Subretinal Fluid (SRF): Quantitative assessment of the maximum cross-sectional area of subretinal fluid at the foveal level.

**(f)** Ellipsoid Zone (EZ) Disruption: Measurement of the length of discontinuity in the hyperreflective EZ band at the foveal level.
